# Silymarin as a feed additive in swine and poultry production: a comprehensive review

**DOI:** 10.1186/s40104-025-01260-3

**Published:** 2025-09-19

**Authors:** Sungbo Cho, Charles Martin Nyachoti, In Ho Kim

**Affiliations:** 1 Department of Animal Biotechnology, Dankook University, Cheonan, 31116 Republic of Korea; 2 Smart Animal Bio Institute, Dankook University, Cheonan, 31116 Republic of Korea; 3 Department of Animal Science, University of Manitoba, Winnipeg, MB R3T 2N2 Canada

**Keywords:** Antibiotic alternatives, Feed additives, Poultry, Silymarin, Swine

## Abstract

The widespread ban on in‐feed antibiotics in many regions has driven the search for natural alternatives to maintain health and production efficiency in swine and poultry. Phytogenic feed additives (PFAs) derived from herbs and plant extracts have emerged as promising candidates owing to their antioxidant, anti‐inflammatory, and antimicrobial properties. Among these, silymarin—a flavonolignan complex extracted from milk thistle (*Silybum marianum*)—has attracted particular attention due to its hepatoprotective and growth‐promoting activities. This review summarizes the chemical characteristics and mechanisms of action of silymarin/silybin. Also, evidence from both experimental and field studies shows that silymarin improves growth performance, nutrient digestibility, gut health, and reproductive outcomes. Advances in formulation technologies, such as micellization, have been addressed for improved bioavailability of silymarin. Despite these promising results, further long-term field studies and economic evaluations are needed to fully integrate silymarin into commercial animal production systems.

## Introduction

The ever‐increasing global demand for livestock products has pushed animal production systems to intensify, but this intensification has brought challenges related to animal health, productivity, and public safety. Traditionally, in‐feed antibiotics were used extensively to improve growth and prevent disease in swine and poultry; however, concerns about the emergence of antibiotic‐resistant bacteria, residual accumulation in the environment, and disturbances in beneficial gut microflora have led to a worldwide shift toward antibiotic-free production systems across species [1–3]. In response, the industry has increasingly turned its attention to natural feed additives derived from plants, known as phytogenic feed additives (PFAs). These compounds, ranging from herbs and spices to essential oils and plant extracts, exhibit a variety of beneficial properties, including antioxidant, anti-inflammatory, antimicrobial, and hepatoprotective effects. Among these natural products, silymarin extracted from the seeds of milk thistle (*Silybum marianum*) has emerged as a particularly promising candidate due to its multifaceted bioactivities. This review examines the current status of PFAs with a special emphasis on silymarin/silybin, focusing on their mechanisms of action and potential applications in swine and poultry production. 

## Challenges in modern swine and poultry production

**Fig. 1:**
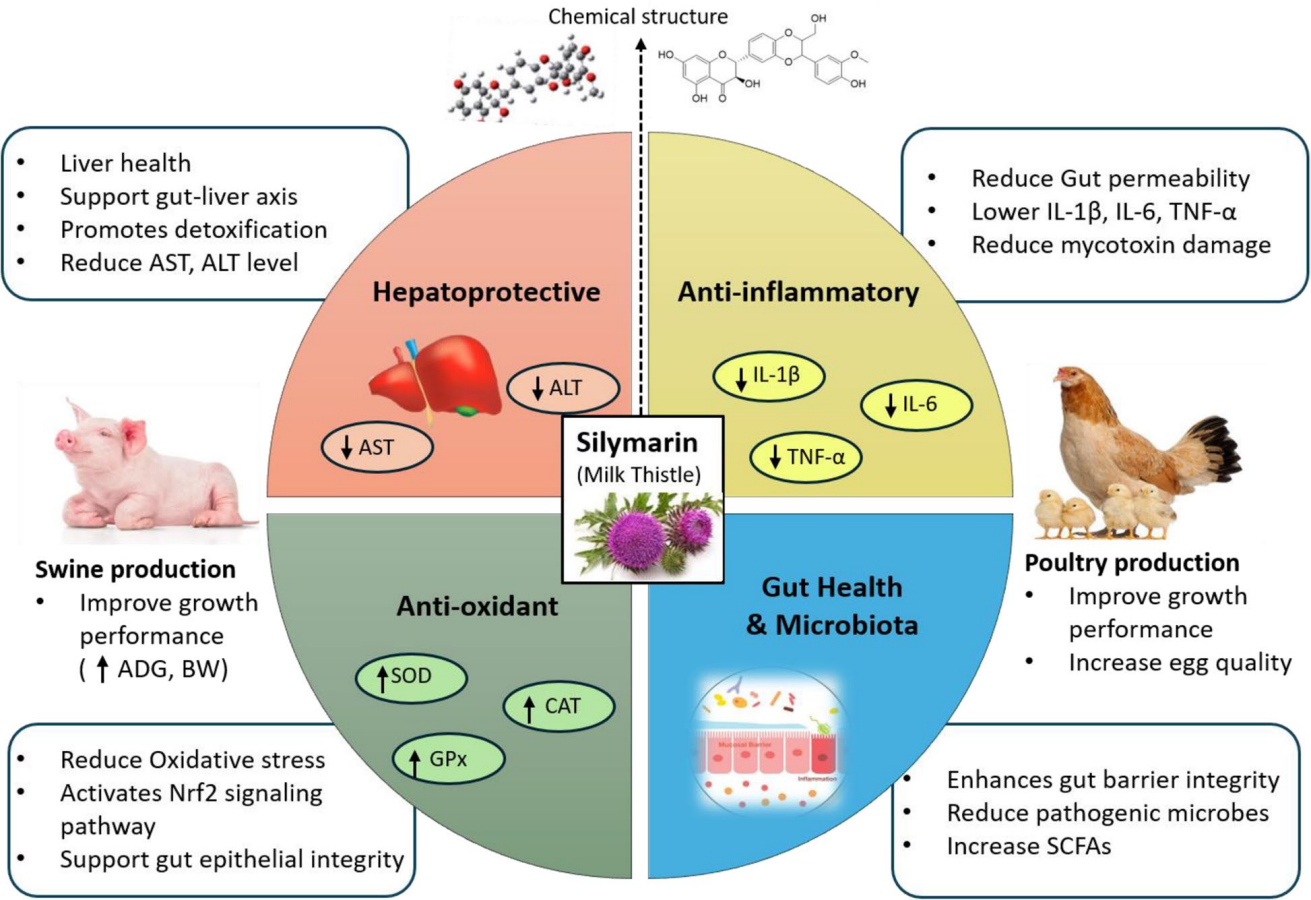
Mechanisms of action of silymarin (milk thistle extract) in swine and poultry nutrition. Silymarin exerts multifactorial biological effects through four primary mechanisms: (1) Antioxidant action via activation of the Nrf2-ARE signaling pathway, leading to increased activity of antioxidant enzymes (SOD, CAT, GPx), enhanced glutathione levels, and reduced oxidative stress markers such as reactive oxygen species (ROS) and malondialdehyde (MDA); (2) Anti-inflammatory effects through inhibition of the NF-κB pathway, resulting in decreased production of pro-inflammatory cytokines including interleukin-1β (IL-1β), interleukin-6 (IL-6), and tumor necrosis factor-alpha (TNF-α); (3) Hepatoprotective functions by modulating hepatic enzymes (e.g., cytochrome P450), stabilizing hepatocyte membranes, enhancing ribosomal RNA synthesis for liver regeneration, and preventing hepatic fibrosis; and (4) Gut health and microbiota modulation, characterized by improved mucin secretion, tight junction integrity, reduced pathogenic bacteria (e.g., *E. coli*, *Salmonella*), increased beneficial microbes (e.g., *Fibrobacteres*, *Actinobacteria*), and elevated production of short-chain fatty acids (SCFAs). These combined effects contribute to improved immune function, and overall growth performance in livestock raised under antibiotic-free systems

Intensive production systems expose animals to a myriad of stressors and physiological challenges that can impair performance. In swine, for example, reproductive efficiency in sows is critical; factors such as inadequate nutritional support during gestation and lactation, oxidative stress from high metabolic demand, and insulin resistance following farrowing can lead to decreased milk production and prolonged wean-to-estrus intervals, thereby reducing overall productivity [4–6]. Moreover, early weaning of piglets induces dramatic changes in intestinal morphology and microbial balance. Rapid reductions in villus height and an unstable gut microbial community compromise nutrient absorption and predispose young pigs to diarrhea and other enteric disorders [7–9]. Similarly, in poultry, the removal of antibiotics has necessitated dietary interventions to maintain growth, gut integrity, and immune status [10]. Antibiotic-free programs in poultry and other livestock can lead to shifts in the gut microbiome (dysbiosis) and a greater risk of enteric diseases such as necrotic enteritis, especially in the absence of antibiotic growth promoters [11]. These challenges have accelerated the search for antibiotic alternatives that not only support growth performance but also improve animal health through the modulation of digestive physiology and the intestinal microbiota (Fig. 1).

## Phytogenic feed additives in non-ruminant nutrition

Phytogenic feed additives comprise a diverse group of naturally occurring compounds produced by plants to defend against pathogens, herbivores, and environmental stresses. Historically, many of these plant-derived compounds have been used in traditional medicine for their health-promoting properties. In the context of animal nutrition, PFAs—including herbs, spices, essential oils, and oleoresins—are now recognized as potential alternatives to in-feed antibiotics. Their modes of action are multifactorial. Many PFAs exhibit strong antioxidant properties by scavenging free radicals and enhancing the activity of endogenous enzymes such as superoxide dismutase and glutathione peroxidase, thereby mitigating oxidative stress [12, 13]. They also display anti-inflammatory properties by modulating cytokine production and inhibiting key inflammatory pathways such as NF-κB [14, 15]. Furthermore, several studies have demonstrated that these additives can improve the secretion of digestive enzymes, enhance bile production, and stimulate intestinal morphology—factors that contribute to improved nutrient digestibility and feed efficiency [16, 17]. While a variety of PFAs such as garlic, thyme, and green tea extract have been widely studied for their effects on animal performance and gut health [10, 12, 17], silymarin exhibits a unique combination of hepatoprotective, antioxidant, and anti-inflammatory activities. Comparative studies indicate that silymarin not only enhances growth performance and nutrient digestibility but also provides superior protection against toxin-induced liver damage, which is a critical benefit in intensive production systems [18]. In contrast to many phytogenics that act primarily via direct antimicrobial effects or flavor-mediated feed intake improvements, silymarin’s strengths lie in its liver-protective and antioxidant effects, which can be especially advantageous during mycotoxin exposure or other metabolic challenges. This unique profile may offer an advantage over other PFAs.

## Chemical characteristics and mechanisms of action of silymarin

Among the array of PFAs, silymarin has garnered significant attention due to its well-documented pharmacological properties. Silymarin is a complex mixture of flavonolignans extracted from the seeds of milk thistle (*Silybum marianum*), with silibinin (also known as silybin) constituting approximately 50%–60% of the complex [19, 20]. Silymarin has been recognized for its robust hepatoprotective, antioxidant, and anti-inflammatory activities. Silymarin exerts its biological effects through multiple interconnected mechanisms. Silymarin acts as a potent antioxidant by directly scavenging free radicals and by upregulating the activity of intrinsic antioxidant enzymes, thereby protecting hepatocytes from toxin-induced damage [21–23]. It activates the nuclear factor erythroid 2-related factor 2 (Nrf2) pathway, leading to the transcription of antioxidant response element (ARE)-driven genes​ [24]. In vivo studies have confirmed that silymarin upregulates Nrf2 and downstream cytoprotective enzymes like heme oxygenase-1 (HO-1) in animal tissues [25]. This translates into elevated activities of antioxidant enzymes (superoxide dismutase, catalase, glutathione peroxidase) and increased glutathione availability, with corresponding reductions in oxidative stress biomarkers such as malondialdehyde (MDA) in pigs. Collectively, by maintaining cellular redox homeostasis via Nrf2 activation, silymarin protects tissues from oxidative damage.

Silymarin also exhibits pronounced anti-inflammatory activity by modulating key signaling pathways. For instance, it is known to inhibit the nuclear factor kappa B (NF-κB) pathway, and reduce the production of pro-inflammatory cytokines, as demonstrated in animal models of liver injury [14, 15]. In various models, silymarin or its active component silibinin down-regulated NF-κB activation and reduced the production of inflammatory cytokines such as tumor necrosis factor-α (TNF-α), interleukin-1β (IL-1β), and IL-6 [24]. For example, in piglets and sows, dietary silymarin supplementation lowered circulating TNF-α and IL-1β levels and was associated with reduced NF-κB activity in intestinal tissues [25–28]. Notably, by inhibiting the NF-κB, silymarin helps break the cycle of inflammation and oxidative stress, as these processes are often linked in livestock under stress or disease conditions.

Moreover, Silymarin is best known as a hepatoprotective agent, and this is highly relevant in farm animals given the liver’s role in metabolism and detoxification and antifibrotic action. At the cellular level, silymarin modulates hepatic enzyme systems and protects liver cell organelles. It has been shown to influence cytochrome P450 enzymes, for instance by decreasing the activity of certain ROS-producing cytochrome reductases that contribute to toxin activation and oxidative stress [29]. By tempering phase I metabolism and enhancing phase II detoxification, silymarin reduces the formation of harmful metabolites. Silymarin also stabilizes cell membranes [30] and promotes ribosomal RNA synthesis in the liver, which supports hepatic cell regeneration and protein synthesis [28]. Additionally, silymarin exerts antifibrotic effects by inhibiting the transformation of hepatic stellate cells into collagen-producing myofibroblasts, thus preventing the progression of liver fibrosis [18]. Together, these hepatoprotective mechanisms explain why silymarin supplementation often lowers liver enzyme release (e.g., ALT, AST) and improves liver histopathology in livestock trials, especially under toxin exposure or metabolic stress conditions.

Lastly, silymarin supplementation helps maintain or increase intestinal mucin production and supports the physical barrier function of the gut. Studies with disease challenged birds have shown that silymarin moderates goblet cell hyperplasia and mucous layer changes: for example, *E. coli*-challenged broiler chicks had an excessive increase in goblet cell counts (a response to infection), which was normalized by dietary silymarin, indicating a healthier mucosal state [31]. Silymarin’s anti-inflammatory action in the gut likely contributes to this effect by reducing local TNF-α and IL-1β, it prevents inflammation-induced breakdown of tight junction proteins. Also, silymarin can influence the composition of the gut microbiome. Silymarin does not have broad antimicrobial activity like antibiotics, but it appears to inhibit certain pathogenic bacteria and favor beneficial microbes in the gastrointestinal tract. For instance, silymarin supplementation in *E. coli-*infected broilers significantly reduced counts of pathogenic bacteria (*E. coli, Salmonella, Klebsiella* and other Gram-negatives) in the ileum, comparable to the effects of an antibiotic, while improving beneficial microbial populations and diversity [31]. Subsequently, dietary silymarin reduced pathogenic bacterial counts in the gut to a degree comparable to an antibiotic treatment**.** In sows, a recent trial using high-throughput 16S rRNA sequencing showed that dietary silymarin altered the fecal microbiota profile: it increased the relative abundance of potentially beneficial phyla such as Fibrobacteres and Actinobacteria while reducing others like Spirochaetes that are often associated with poor gut health [28]. The studies suggest silymarin has prebiotic-like effects, enhancing lactate-utilizing and fiber-fermenting bacteria. Modulation of the gut microbiota by silymarin could thus lead to greater production of beneficial metabolites like butyrate and less accumulation of toxins or deleterious metabolites in the gut. Notably, in a recent murine study, silibinin increased the intestinal production and absorption of butyrate by upregulating butyrate-producing microbes and transporters [32]. This microbiota-centered mechanism complements the direct antioxidant and anti-inflammatory actions of silymarin, ultimately contributing to improved gut health and immunity in swine and poultry. These metabolic actions of silymarin have been linked to improved biochemical markers in both experimental and field studies (Table 1).

**Table 1:** Efficacy of dietary silymarin in monogastric animals

Animals	Trial period	Dose level	Effects	Source
Laying hen	20 weeks	0, 200, and 400 mg/kg silymarin	Significantly increased dry matter digestibiliy and reduced AST, ALT, serum triglycerides and total cholesterol. In addition, improved egg laying rate and feed conversion ratio	[53]
Hyline brown Laying hen	12 weeks	0–0.06% Micelle silymarin	Increased egg quality by decreasing the downgraded egg. Also, increased silymarin absorption in blood and reduced serum AST, ALT, lactate dehydrogenase, triglyceride, and cholesterol	[44]
Ross 308 broiler	6 weeks	0, 0.24, and 0.36 g/d/animal milk thistle extract in drinking water	Increased final BW, BWG, antioxidant capacity in the serum and in pectoral muscle and reduced the foot lesion score. Significant differences were found in the proportion of abdominal fat	[43]
Weaning pig	6 weeks	0.05% to 0.10% Micelle silymarin extract	Boost ADG by improving ADFI	[54]
Lactating sow	Post farrowing to 21 days of lactation	1 or 8 g/d silymarin plant extract	Neither significant nor adverse effect found on body weight, lactation feed intake, backfat, prolactin concentrations or oxidative status in sows	[6]
Lohmann Pink laying hens	12 weeks	250, 500, 750, or 1,000 mg/kg silymarin	Silymarin supplementation increased ADFI and reduced feed-to-egg ratio; AST, ALT, serum triglycerides and total cholesterol in hens. Also, liver enzyme expressions influenced (FASN, ACC, Apo-VLDL II, FXR, and CYP7A1) by altering the cecal microbiota composition	[55]
Growing pig	6 weeks	0, 0.025%, 0.050%, 0.10% MS	MS supplementation had tended to increase ADG and ADFI, and linearly increased nutrient absorption. Also, it showed linearly increased *Lactobacillus* and reduced *Escherichia coli* count. MS supplementation had higher absorption rate in the blood with micelle form (after the 1^st^, 2^nd^, 4^th^, 8^th^, 12^th^ and 24^th^ hour of feeding)	[36]
Ross 308 broiler	6 weeks	0, 500, and 1,000 mg/kg silymarin	Silymarin supplement had linearly increased ADG, ADFI, and FCR; Decreased ileal microflora by modifying goblet cell count and lamina propria lymphoid follicle numbers (LLFN). Also, increased villi height and VH:CD ratio and reduced crypt depth	[31]
Ross 308 broiler	6 weeks	0/2% and 2/3% ground milk thistle seeds (GMTS)	GMTS had increased BW by 3% and decreased FCR by 7%, highest content of polyunsaturated fatty acids (PUFA) in the breast (38.06%) and leg (37.63%) muscles. Decrease lightness color (L*), and WHC of breast muscle	[42]
Sow and offspring	108^th^ day of gestation to weaning	40 g/d silymarin	Improved ADG and weaning weight in offspring; Also, increased catalase (CAT) on day 18 of lactation and glutathione peroxidase (GSH-Px) on day 7 of lactation in sow. Reduce TNF-α on day 7 of lactation and IL-1β on day 18 of lactation	[38]
Hyline brown Laying hen	12 weeks	0–0.06% Micelle silymarin	MS supplements significantly reduced AST, ALT, lactate dehydrogenase, triglyceride, and cholesterol. Increased egg quality parameter and decreased the downgraded egg	[46]
Sow	6 weeks	Basal diet plus 250 and 500 mg/kg Silymarin	Incorporating silymarin to sow diet reveal increased total feed intake, urea content in regular milk, decreased fecal isobutyric acid concentration. Also showed tended to increase total antioxidant capacity, triglyceride, reduced serum total protein and albumin	[57]
Ross 308 broiler	5 weeks	0, 250, and 500 mg/kg Aflatoxin B_1_ (AFB1) + 0, 0.5%, and 1.0% silymarin	Silymarin alone or in combination with AFB1 resulted in significant changes in carcass weight and internal organs	[58]
Cobb 500 broiler	6 weeks	0 (Control), 160, 200, 240, 280 and 320 mg/kg silymarin	Increased FI and FCR, increased humoral immunity	[56]
Cobb 400 broiler	6 weeks	CON + 500 mg/kg MS; CON + 400 µg/kg CP; CON + MS + chromium picolinate (CP)	Increased BW, ADG, ADFI, and FCR were observed in chicks fed combination of MS and CP diet	[40]
Ross 508 broiler	60 d	0, 40, and 80 mg/kg silymarin (dry extract)	Reduce lipid content in breast and thigh muscles and increased resistance to oxidative stress with no adverse effect on growth performance	[41]
Ross 308 broiler	5 weeks	0, 0.02%, 0.04%, and 0.06% Micelle silymarin	Linearly increased body weight, DM, N and E digestibility, reduced the methyl and acetic acid emission, increased cecal *Lactobacillus* counts, WHC, breast muscle and bursa of fabricius weight, reduce drip loss on d 1, 3, 5, and 7	[34]
Sow	108^th^ day of gestation to weaning	40 g/d silymarin	Decreased pro-inflammatory cytokine IL-1β on the 18^th^ day of lactation, tended to decrease somatic cell count. Reduced the gut bacterial community and the richness of the gut microbial community. Increase the relative abundance of Fibrobacteres and Actinobacteria and tended to reduce the relative abundance of Spirochaetaes and Tenericutes at the phylum level	[28]
Fattening pig	10 weeks	0, 0.05%, 0.1%, and 0.2% Micelle silymarin	Linearly increased ADG, N digestibility, reduced H_2_S and NH_3_ concentrations, increased cecal *Lactobacillus* counts, improved total antioxidant capacity concentration	[35]
Sow and offspring	109^th^ day of gestation to weaning d21	0–0.2% Micelle silymarin	Linearly lactation feed intake, milk yield and reduce BW loss in sows. Increased litter weight ADG in offspring. Reduce AST and increased activity of superoxide dismutase (SOD) at parturition and glutathione peroxidase (GSH-Px), lower oxidized glutathione (GSSG) concentrations, and GSSG/GSH (glutathione) ratio on day 21 of lactation	[37]
Weaning pig	6 weeks	0.05% to 0.10% Micelle silymarin	Linearly increased ADG and ADFI along with increased IgM and glutathione level	[25]

## Applications of silymarin in swine production

Various studies have investigated the role of silymarin in swine production. The dietary inclusion of silymarin has been linked to improved liver function and detoxification, which is particularly important given the exposure of pigs to various toxins. For example, silymarin supplementation protects the liver from mycotoxins such as aflatoxin B_1_ [33] and improves blood enzyme profiles. Several studies have shown that silymarin supplementation can enhance average daily gain (ADG) and improve feed efficiency by protecting the liver from oxidative damage and supporting efficient nutrient metabolism [34–36]. Specifically, one study observed a linear increase in ADG and average daily feed intake (ADFI) in weaning pigs with 0.05% to 0.10% micelle silymarin supplementation [25], while fattening pigs exhibited linear improvements in ADG with dosages up to 0.2% [35]. Additionally, growing pigs fed diets supplemented with 0.05% to 0.10% silymarin consistently showed enhanced growth performance over a 6-week trial [36]. In lactating sows, feeding diets supplemented with silymarin from day 109 of gestation through day 21 of lactation increased feed intake and milk yield while reducing body weight loss, ultimately improving litter performance [37, 38]. Additionally, in lactating sows, silymarin has been associated with reduced body weight loss, increased feed intake, improved milk yield, and enhanced piglet performance, likely due to its capacity to mitigate oxidative stress and modulate inflammatory responses [6, 28]. Silymarin supplementation has also been reported to improve meat quality parameters such as pH, reduced lipid peroxidation, and enhanced water-holding capacity [39].

## Application of silymarin in poultry production

In broiler chickens, similar benefits as those reported for swine have been observed. Dietary silymarin enhances voluntary feed intake, body weight gain, and feed efficiency [40, 41]. For example, Ross 308 broilers fed diets supplemented with silymarin exhibited a linear increase in body weight and ADG [42, 43], and Cobb 400 broilers showed further improvements when silymarin was combined with chromium picolinate [40]. Studies have demonstrated that, in Ross 308 broilers, supplementation with silymarin increased nutrient digestibility specifically, dry matter (DM), nitrogen (N), and energy (E) digestibility in a linear fashion [34]. Silymarin supplementation in poultry not only improves growth performance and nutrient digestibility but also contributes to better meat quality through reduced lipid peroxidation and enhanced water-holding capacity [41–43]. Finally, improvements in egg production and enhanced egg quality (i.e., improved Haugh unit, albumen height, shell strength, and shell thickness) have been reported as a result of feeding silymarin-supplemented diets [42, 43].

## Additional effects of silymarin in swine and poultry production

Beyond growth performance and nutrient digestibility, silymarin has been shown to reduce fecal gas emissions. For instance, in fattening pigs, dietary supplementation with silymarin reduced hydrogen sulfide and ammonia levels in feces [35], and in broilers, silymarin reduced emissions of methyl mercaptan (methanethiol) and acetic acids [34]. Silymarin also beneficially modulates gut microbiota. In growing pigs, it increased *Lactobacillus* counts while reducing *Escherichia coli* populations [36]. In broilers, similar improvements in cecal *Lactobacillus* counts have been observed [34], and high dosages (500–1,000 mg/kg) in Ross 308 broilers decreased ileal microflora [31]. Furthermore, silymarin improves immune and antioxidant parameters. In Hyline brown laying hens, supplementation at up to 0.06% reduced serum levels of liver enzymes (AST and ALT), lactate dehydrogenase, triglycerides, and cholesterol [44–46]. In weaning pigs, silymarin increased immunoglobulin M (IgM) and glutathione levels [25], and in sows, supplementation reduced pro-inflammatory cytokine IL-1β and improved antioxidant enzyme activities [28, 38]. Finally, safety is a crucial consideration for any feed additive. Silymarin is generally regarded as safe, with a low toxicity profile reported in both animal studies and clinical trials. Saller et al*.* [47] and Polyak et al. [48] have documented that even at high doses, silymarin does not produce significant adverse effects. In livestock studies, long-term supplementation of silymarin has not been associated with any negative impacts on growth, feed intake, or overall health [6, 28]. These findings support the potential for silymarin to be safely incorporated into animal diets.

## Economic feasibility and cost-effectiveness

From an economic standpoint, silymarin appears to be a practical phytogenic additive when considering both its costs and the benefits it delivers in production animals. Milk thistle, the source of silymarin, is grown in many regions worldwide and its seeds (and by-products from supplement or pharmaceutical industries) are relatively inexpensive. In fact, one review emphasizes that milk thistle extracts can be used as a “*non-toxic, safe, and cheap*” liver tonic feed additive, providing a natural substitute for synthetic drugs in poultry diets [49]. Compared to some essential oils or proprietary botanical blends, silymarin extract is cost-effective on a per-dose basis, especially given the low inclusion rates (often 0.05% to 0.1% of diet) that have shown efficacy. When silymarin improves feed intake and growth, it can enhance feed conversion ratios (FCR), meaning more weight gain per unit of feed—a direct economic gain. For instance, broilers challenged with *E. coli* and supplemented with silymarin not only grew faster but also had significantly better FCR throughout the rearing period [31]. Improved FCR translates to feed cost savings, which is a major component of production expenses. Similarly in pigs, trials have noted higher average daily gain and nutrient digestibility in silymarin-fed groups [25, 27]. These performance boosts can shorten the time to market or reduce feed required for a given body weight, improving profitability. However, the practical application of silymarin in commercial livestock production also depends on economic considerations. High-quality silymarin formulations (e.g., purified or micellized silymarin) may be more costly than some conventional additives, but the potential benefits such as improved animal health, reduced incidence of toxin-related losses, and decreased reliance on antibiotics, may compensate for these costs. Integrating silymarin into livestock production systems can lead to overall production cost savings by improving feed efficiency and animal productivity. While crude milk thistle seed meal is cheap, investing in advanced delivery forms like nano- or micelle-silymarin could yield higher returns via better bioavailability. Therefore**,** there is a cost-benefit tradeoff, which indicates the added manufacturing cost of enhanced formulations must be weighed against the gains in efficacy. Preliminary data indicate that the improvements in growth rate and feed conversion with micellized silymarin can offset the higher additive cost when feed prices and output value are accounted for. Nonetheless, detailed economic analyses and long-term field trials are necessary to fully elucidate the cost-effectiveness of silymarin-based supplements under commercial conditions.

## Challenges and future perspectives

Despite promising results, the use of PFAs—including silymarin—faces several challenges. Inconsistencies in results across studies may be due to differences in extraction methods, active compound concentrations, diet composition, and animal breed or age [50–52]. Silymarin’s poor water solubility remains a challenge for its bioavailability. Technological approaches like micellization have been proposed and tested to improve its absorption [39]. Indeed, piglets fed micelle-formulated silymarin show significantly higher plasma silybin levels and greater performance improvements compared to those receiving conventional silymarin. Such formulation advances accelerate the beneficial roles of silymarin such as hepatoprotective, antiinflammation, and antioxidant. In addition, standardized formulations and dosing regimens are still lacking, which complicates comparisons between studies and the translation of experimental findings to commercial practice. Finally, the practical application of silymarin in commercial livestock production also depends on economic considerations. Although basic silymarin seed extracts are inexpensive, high-purity or enhanced-bioavailability products can be more costly, as noted above. However, the potential benefits such as improved animal health, reduced incidence of disease or toxin-related losses, and decreased need for therapeutic interventions may compensate for these costs when evaluated at the system level. To effectively replace in-feed antibiotics, a cocktail of strategies, combining various PFAs, acids, probiotics, etc., is likely most beneficial. There is a need to investigate the potential synergistic effects of silymarin with other feed additives. For example, its combination with chromium picolinate or with probiotics has shown additive benefits [40]. Future research should explore such combinations, optimal inclusion rates, and the consistency of responses across different production conditions. 

## Conclusion

The integration of silymarin as a phytogenic feed additive holds considerable promise for enhancing the sustainability of antibiotic‐free production systems in both swine and poultry. Silymarin’s robust hepatoprotective, antioxidant, and anti‐inflammatory activities have been demonstrated to improve growth performance, nutrient digestibility, reproductive outcomes, and meat quality, while also favorably modulating gut health and immune responses. Advances in formulation strategies, such as micellization, have begun to overcome the inherent limitations of silymarin’s poor water solubility and limited bioavailability, ensuring that its bioactive components can effectively reach target tissues. Comparative analyses show that silymarin is unique, particularly in its ability to counteract toxin-induced liver damage gives it an advantage over other phytogenic feed additives. Safety studies consistently report a low toxicity profile even at high doses, and preliminary economic analyses suggest that the improved feed efficiency and animal productivity associated with silymarin supplementation could offset the cost of its advanced formulations. However, to fully realize the potential of silymarin in commercial production, further long-term field studies, comprehensive economic evaluations, and detailed investigations into its cellular and molecular mechanisms are warranted. Ultimately, the incorporation of a well-characterized silymarin into livestock diets is poised to contribute significantly to more sustainable and effective animal production systems in the post-antibiotic era.

## Data Availability

Not applicable.

## Abbreviations

ADFI: Average daily feed intake
ADG: Average daily gain
AFB1: Aflatoxin B_1_
ALT: Alanine transaminase
AST: Aspartate transferase
BW: Body weight
CAT: Catalase
CD: Crypt depth
CYP7A1: Cholesterol 7a-hydroxylase
DM: Dry matter
E: Energy
FCR: Feed conversion ratio
GCC: Goblet cell count
GSH-Px: Glutathione peroxidase
GSSG: Oxidized glutathione
IgM: Immunoglobulin M
IL-1β: Interleukin-1 beta
LLFN: Lymphoid follicle numbers
MS: Micelle silymarin
N: Nitrogen
PFAs: Phytogenic feed additives
PUFA: Polyunsaturated fatty acids
SOD: Superoxide dismutase
TNF-α: Tumor necrosis factor-alpha
VH: Villus height
WHC: Water holding capacity
